# The prevalence of HIV pre-exposure prophylaxis (HIV-PrEP) use and HIV-PrEP-to-need ratio in nine Canadian provinces, 2018–2021

**DOI:** 10.14745/ccdr.v51i01a05

**Published:** 2025-01-02

**Authors:** Nashira Popovic, Qiuying Yang, Laurence Campeau, Janelle Elliott, Anson Williams, Viviane D Lima, Paul Sereda, Joseph Cox

**Affiliations:** 1 Centre for Communicable Disease and Infection Control, Public Health Agency of Canada, Ottawa, ON; 2 BC Centre for Excellence in HIV/AIDS, Vancouver, BC; 3 Department of Medicine, University of British Columbia, Vancouver, BC; 4 Department of Epidemiology, Biostatistics and Occupational Health, McGill University, Montréal, QC

**Keywords:** HIV, pre-exposure prophylaxis (PrEP), epidemiology, prevention, HIV-PrEP use, HIV-PrEP-to-need ratio (PnR)

## Abstract

**Background:**

Measuring trends in HIV pre-exposure prophylaxis (HIV-PrEP) uptake is important to inform planning for prevention programs and policies. The HIV-PrEP-to-need ratio (PnR) is a construct used by public health organizations to explore disparities in the provision of HIV-PrEP across geographic areas and demographic categories (e.g., age, sex).

**Methods:**

This is a retrospective database review study using administrative pharmacy data, containing limited demographic information, from nine Canadian provinces. Annual estimates of persons taking HIV-PrEP and PnR were generated using data from the company IQVIA and the BC Centre for Excellence on HIV/AIDS. Data on new HIV diagnoses were obtained from the National HIV Surveillance System. The PnR was defined as the number of HIV-PrEP users divided by the number of new HIV diagnoses annually and is interpreted as the number of HIV-negative people using HIV-PrEP each year for every person newly diagnosed with HIV.

**Results:**

In 2021, an estimated 23,644 individuals were prescribed HIV-PrEP, corresponding to an HIV-PrEP prevalence of 66.9 per 100,000 persons. This represents a 1.8-fold increase since 2018. The overall PnR was 16.8, meaning that for every person newly diagnosed with HIV, 17 HIV-negative individuals were taking HIV-PrEP. There were disparities between provinces (PnR range: 1.5/100,000–37.7/100,000) and between males and females (PnR 22.6 and 1.2, respectively). Females, individuals aged 0–19 years, and those in Manitoba, Saskatchewan and Prince Edward Island, had lower levels of HIV-PrEP use relative to epidemic need.

**Conclusion:**

In Canada, the use of HIV-PrEP increased from 2018 to 2021 and uptake varied by age, sex and province. HIV-PrEP-to-need ratio is a useful measure to assess uptake of HIV-PrEP as a prevention strategy and could be used to explore disparities in provision across provinces and available demographic categories. However, PnR could be improved with more information on key populations and other attributes, such as race/ethnicity, socioeconomic status and residence of city/rural area.

## Introduction

HIV pre-exposure prophylaxis (HIV-PrEP) is highly effective and has the potential to make a significant contribution to reducing Canada’s HIV incidence ((1)). In 2016, Health Canada approved the drug combination tenofovir disoproxil fumarate/emtricitabine (TDF/FTC) for use as HIV-PrEP, and in July 2017, lower-cost generic versions became available in Canada ((1,2)).

The Government of Canada has endorsed global health sector strategies on HIV, viral hepatitis and sexually transmitted infections for the period of 2022–2030. This includes ensuring continued engagement of people living with HIV in treatment and care services and leveraging innovations, such as new treatment regimens and new prevention approaches ((3–6)). Canadian National Surveillance data shows that new HIV diagnoses have been decreasing for several years ((7)) and mathematical modelling suggests that HIV incidence is decreasing overall in Canada ((8)). The estimated annual number of new HIV infections in Canada has decreased from about 4,000 per year in the mid-1980s to around 2,000–2,500 in the 2000s, following the introduction of antiretroviral therapy (ART), with a further decrease to 1,520 in 2020 ((8)). Although HIV incidence appears to be declining nationally, this overall trend does not account for the heterogeneity in HIV infections across Canada, as incidence appears to be increasing within some jurisdictions.

Previous studies showed that when adherence is maintained, daily HIV-PrEP use reduced HIV transmission by 36% to 99% in people who inject drugs (PWID), heterosexual individuals, and gay, bisexual, and other men who have sex with men (gbMSM) ((9–12)). Murchu *et al.* ((13)) conducted a systematic review and meta-analysis of randomized controlled trials of the effectiveness and safety of oral HIV-PrEP to prevent HIV. They found that HIV-PrEP is effective in gbMSM (RR 0.25; 95% CI: 0.1–0.61) and PWID (RR 0.51; 95% CI: 0.29–0.92), but not in heterosexuals (RR 0.77; 95% CI 0.46–1.29).

Reducing new HIV infections by 2030 will require multi-pronged strategies to support combination prevention, including condom promotion and educational programs ((14)), testing and the use of both post-exposure prophylaxis (PEP) and HIV-PrEP in high-risk populations.

HIV-PrEP-to-need ratio (PnR) is defined as the ratio of HIV-PrEP users per new HIV diagnoses. A higher level of PnR indicates more HIV-PrEP users relative to estimated need ((15)). Tan *et al.* (2021) (16) found that PnRs were highest in those 30–39 years of age, males, Toronto and the Central East and West regions of Ontario. Siegler *et al.* (2020) (15) found that Medicaid expansion and HIV-PrEP drug assistance programs in the United States were associated with higher HIV-PrEP use in states that adopted those policies, after controlling for potential confounders. Thus, to reduce HIV-PrEP disparities, public health strategies must be developed to reach those most in need, especially historically disadvantaged communities ((17)). These studies suggested that PnR is useful for future assessments of HIV prevention strategy uptake.

This study updated a previous analysis of HIV-PrEP uptake in Canadian provinces ((2)), and estimated HIV-PrEP-use prevalence and PnR for nine Canadian provinces from 2018–2021, by sex, age group and province. This information could be used to identify groups and populations with lower HIV-PrEP uptake, or higher HIV-PrEP need, thus informing policymakers and program planners.

## Methods

### Prevalence of HIV-PrEP users

**Figure 1:**
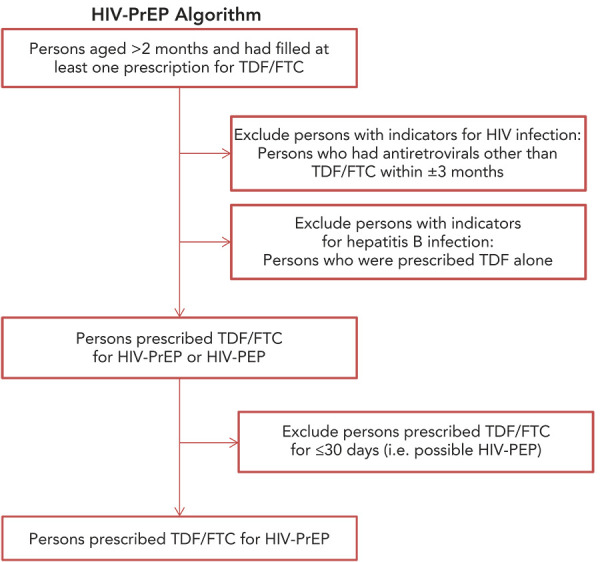
Algorithm to assign HIV-PrEP treatment indication Abbreviations: HIV-PrEP, pre-exposure prophylaxis; PEP, post-exposure prophylaxis; TDF/FTC, tenofovir disoproxil fumarate/emtricitabine

Annual estimates of persons using HIV-PrEP in Canada were generated for 2018–2021 from a prescription database held by the company IQVIA. A validated algorithm ((18)) was used to distinguish users of TDF/FTC for HIV-PrEP from those using TDF/FTC for HIV or hepatitis B treatment or post-exposure prophylaxis (**Figure 1**). The algorithm was adapted from the validated United States Centers for Disease Control algorithm ((18,19)) and modified to fit the Canadian context ((2)).

Briefly, in a given year, persons older than two months of age who had one or more TDF/FTC prescriptions were included. Since TDF/FTC is also used to treat HIV or hepatitis B infections and as HIV PEP, several exclusion criteria were applied: 1) persons who were prescribed antiretrovirals other than TDF/FTC within ±3 months (persons on HIV treatment); 2) persons who were prescribed with TDF alone (for hepatitis B treatment); and 3) persons who were prescribed TDF/FTC for less than or equal to 30 days (PEP users). In any given year, persons prescribed TDF/FTC who were not excluded with our algorithm were considered HIV-PrEP users.

All ages were taken into account when IQVIA extracted the data and estimated the number of projected patients by indication; however, the results for patients younger than 15 years of age were omitted due to small counts. Prevalence of HIV-PrEP users was defined as the number of HIV-PrEP users in a calendar year, divided by the total population in that year. It is expressed as HIV-PrEP users per 100,000 population.

### Data sources

Data on new HIV diagnoses were obtained from the National HIV Surveillance System ((7)). These data include only people diagnosed with HIV for the first time in Canada and do not include individuals who were previously diagnosed with HIV in another country and then emigrated to Canada.

Data on antiretroviral drug prescriptions dispensed in eight provinces (Manitoba [MB], Ontario [ON], New Brunswick [NB], Newfoundland and Labrador [NL], Nova Scotia [NS], Prince Edward Island [PE], Québec [QC] and Saskatchewan [SK]) between January 1, 2018, and December 31, 2021, were extracted by IQVIA from the company’s geographical prescription monitor dataset. Data from Alberta (AB) are not included in IQVIA’s dataset, since coverage within this province does not meet the threshold for reporting projected patient counts. The HIV-PrEP use number in British Columbia (BC) was provided by the BC Centre for Excellence in HIV/AIDS (BC-CfE) ((20)). These nine provinces represented 88.1% of the Canadian population in 2021. Population size estimates were obtained from Statistics Canada ((21)).

The IQVIA database includes Canadian aggregate dispensed prescription data projected from a sample of approximately 6,000 pharmacies in eight provinces, representing close to 60% of all retail pharmacies in Canada. Patient counts were then projected from this sample of pharmacies to extrapolate for the entire province.

In January 2018, BC implemented an HIV-PrEP program as part of a comprehensive Treatment as Prevention strategy, within which BC residents are eligible to receive publicly funded HIV-PrEP via the BC-CfE HIV-PrEP program. The BC-CfE HIV-PrEP program database is a centralized clinical registry, which stores data from various sources relating to demographic and behavioural information, clinical outcomes (laboratory results) and antiretroviral medication dispensation data ((20)).

### HIV-PrEP-to-need ratio (PnR)

HIV-PrEP-to-need ratio was defined as the ratio of the number of HIV-PrEP users to the number of people newly diagnosed with HIV in the same year ((15,16,19)). New HIV diagnoses were used as an epidemiological proxy for HIV incidence from 2018–2021. HIV-PrEP-to-need ratio was used to describe HIV-PrEP coverage overall and per province and demographic subgroups (sex and age group) relative to new HIV diagnoses in the same year. The PnR attempts to assess and compare how well-targeted HIV-PrEP coverage is to the groups and populations that can benefit from it the most and can be understood as the number of people using HIV-PrEP each year for every person newly diagnosed with HIV. A PnR of 2.0 means that for every person newly diagnosed with HIV in a year, two HIV-negative people were using HIV-PrEP.

### Analyses

The two outcomes (HIV-PrEP uptake and PnR) were calculated for nine Canadian provinces from 2018–2021 and stratified by sex, age group and province over this time period. Chi-square tests were performed among sex, age groups and provinces. Cochran-Armitage trend tests were conducted to determine whether HIV-PrEP prevalence and PnR changed significantly over time. Analyses were performed using SAS Enterprise Guide 7.1 (SAS Institute).

## Results

### Overall trends

**Figure 2:**
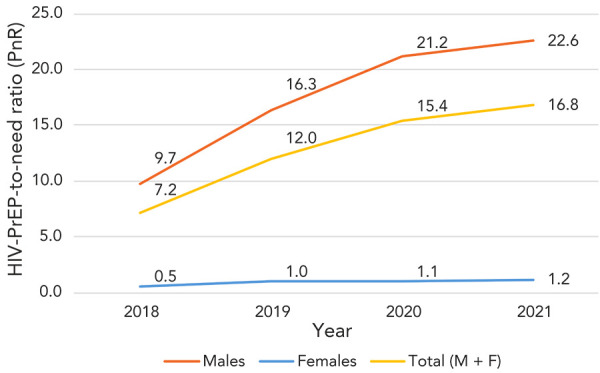
HIV-PrEP-to-need ratio by sex in nine Canadian provinces, 2018–2021 Abbreviations: F, females; M, males; HIV-PrEP, pre-exposure prophylaxis; PnR, HIV-PrEP-to-need ratio

In 2021, a total of 23,644 individuals were estimated to be on TDF/FTC for HIV-PrEP in nine Canadian provinces (BC, MB, ON, NL, NB, NS, PE, QC and SK), resulting in an estimated HIV-PrEP prevalence of 69.9 per 100,000 persons. The estimated number of HIV-PrEP users increased over the four-year period (**Table 1**), showing a 1.8-times increase from 13,222 in 2018 to 23,644 in 2021 (*p* trend<0.001). The PnR was 16.8 in 2021, meaning that for every person newly diagnosed with HIV, 17 HIV-negative individuals were using HIV-PrEP (Table 1). From 2018–2021, annual HIV-PrEP use prevalence increased while reported HIV incidence declined, leading to a 2.3-times increase in PnR (*p* trend<0.001) (Table 1, **Figure 2**).

**Table 1:** HIV-PrEP users and HIV-PrEP-to-need ratio by year in nine Canadian provinces, for both sexes

Year	HIV-PrEP use		New HIV diagnoses		PnR
	Count	Prevalence (n/100,000)	Count	Rate of new HIV diagnoses (n/100,000)	
2018	13,222	40.3	1,839	5.6	7.2
2019	19,689	59.1	1,646	4.9	12.0
2020	20,771	62.0	1,351	4.0	15.4
2021	23,644	69.9	1,406	4.2	16.8

Abbreviations: HIV-PrEP, pre-exposure prophylaxis; PnR, HIV-PrEP-to-need ratio

### Trends by sex

HIV-PrEP use was much greater among males than females, with almost all (98.0%) HIV-PrEP users being males during the four-year period (*p* chi-square<0.001). In 2021, the PnR for males was 22.6, meaning that for every male newly diagnosed with HIV, 23 HIV-negative males were using HIV-PrEP. Among males, the number of HIV-PrEP users was 1.8 times higher in 2021 than in 2018 (*p* trend<0.001), HIV-PrEP use prevalence was 1.7 times higher in 2021 than in 2018 (*p* trend<0.001) and PnR was 2.3 times higher in 2021 than in 2018 (*p* trend<0.001) (Figure 2, **Table 2**).

**Table 2:** HIV-PrEP users and HIV-PrEP-to-need ratio by year in nine Canadian provinces, males only

Year	HIV-PrEP use		New HIV diagnoses		PnR
	Count	Prevalence (n/100,000)	Count	Rate of new HIV diagnoses (n/100,000)	
2018	12,947	79.6	1,335	8.2	9.7
2019	19,234	116.4	1,178	7.1	16.3
2020	20,351	122.5	962	5.8	21.2
2021	23,195	138.1	1,028	6.1	22.6

Abbreviations: HIV-PrEP, pre-exposure prophylaxis; PnR, HIV-PrEP-to-need ratio

In 2021, the PnR for females was 1.2. Among females, the number of HIV-PrEP users was 1.6 times higher in 2021 than in 2018 (*p* trend<0.001), HIV-PrEP use prevalence was 1.5 times higher in 2021 than in 2018 (*p* trend<0.001) and PnR was 2.4 times higher in 2021 than in 2018 (*p* trend<0.001) (**Table 3**, Figure 2).

**Table 3:** HIV-PrEP users and HIV-PrEP-to-need ratio by year in nine Canadian provinces, females only

Year	HIV-PrEP use		New HIV diagnoses		PnR
	Count	Prevalence (n/100,000)	Count	Rate of new HIV diagnoses (n/100,000)	
2018	275	1.7	504	3.0	0.5
2019	455	2.7	468	2.8	1.0
2020	420	2.5	389	2.3	1.1
2021	449	2.6	378	2.2	1.2

Abbreviations: HIV-PrEP, pre-exposure prophylaxis; PnR, HIV-PrEP-to-need ratio

### Trends by age

**Figure 3:**
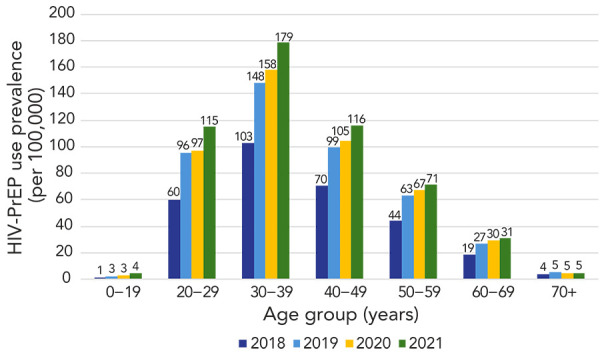
Annual HIV-PrEP use prevalence (per 100,000 persons) by age group, nine Canadian provinces, males and females, 2018–2021 Abbreviation: HIV-PrEP, pre-exposure prophylaxis

**Figure 4:**
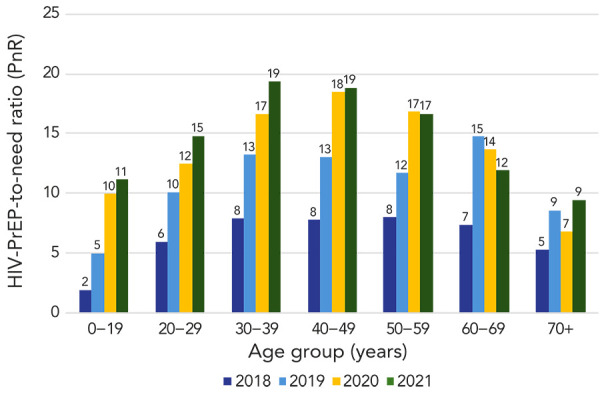
HIV-PrEP-need ratio by age group, males and females, nine Canadian provinces, 2018–2021 Abbreviation: HIV-PrEP, pre-exposure prophylaxis

In 2021, HIV-PrEP use and PnR were highest among people aged 30–39 years (HIV-PrEP users: 8,337; HIV-PrEP use prevalence: 179.1/100,000; PnR: 19.3) and were lowest among individuals aged 0–19 years and 70+ years (*p* chi-square<0.001) (**Table 4**). Between 2018–2021, the annual prevalence of HIV-PrEP use increased among all age groups (*p* trend<0.01) and the PnR increased in all age groups (*p* trend<0.01), except those aged 60–69 years (*p* trend=0.11) (**Figure 3** and **Figure 4**).

**Table 4:** HIV-PrEP users and HIV-PrEP-to-need ratio by age group, nine Canadian provinces, 2021

Age group (years)	HIV-PrEP use		New HIV diagnoses		PnR
	Counts	Prevalence (n/100,000)	Counts	Rate of new HIV diagnoses (n/100,000)	
0–19	301	4.3	27	0.4	11.1
20–29	5,216	115.2	352	7.8	14.8
30–39	8,337	179.1	431	9.3	19.3
40–49	4,957	116.0	263	6.2	18.8
50–59	3,250	71.5	195	4.3	16.7
60–69	1,356	31.1	114	2.6	11.9
70+	227	5.1	24	0.5	9.5

Abbreviations: HIV-PrEP, pre-exposure prophylaxis; PnR, HIV-PrEP-to-need ratio

### Geographical trends

**Figure 5:**
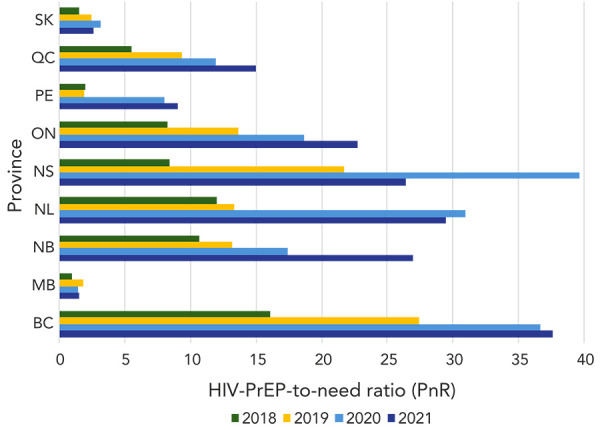
HIV-PrEP-to-need ratio by province, 2018–2021 Abbreviations: BC, British Columbia; HIV-PrEP, pre-exposure prophylaxis; MB, Manitoba; NB, New Brunswick; NL, Newfoundland and Labrador; NS, Nova Scotia; ON, Ontario; PE, Prince Edward Island; QC, Québec; SK, Saskatchewan

Provincial HIV-PrEP use prevalence in 2021 ranged widely from 15.9–107.6 per 100,000 persons (average: 69.9/100,000) (*p* chi-square<0.001). The provincial PnR also ranged widely from 1.5–37.7 (average: 16.8) (*p* chi-square<0.001) (**Table 5**). HIV-PrEP use prevalence in 2021 was the highest in BC, ON, QC and SK; however, given the higher rates of new HIV diagnoses, the PnR was lowest in MB and SK (Table 5). From 2018–2021, patterns of HIV-PrEP use varied. Trend test was significant in all provinces (*p* trend<0.01) except for NL (*p* trend=0.13) and MB (*p* trend=0.05), and decreasing in SK, NS and NL between 2020–2021 (**Figure 5**).

**Table 5:** HIV-PrEP users and HIV-PrEP to need ratio by province, 2021

Province	HIV-PrEP use		New HIV diagnoses		PnR
	Count	Prevalence (n/100,000)	Count	Rate of new HIV diagnoses (n/100,000)	
British Columbia	5,650	107.6	150	2.9	37.7
Manitoba	221	15.9	145	10.5	1.5
New Brunswick	216	27.2	8	1.0	27.0
Newfoundland and Labrador	118	22.6	4	0.8	29.5
Nova Scotia	423	42.3	16	1.6	26.4
Ontario	11,045	74.1	485	3.3	22.8
Prince Edward Island	36	21.7	4	2.4	9.0
Québec	5,307	61.5	354	4.1	15.0
Saskatchewan	628	53.2	240	20.3	2.6

Abbreviations: HIV-PrEP, pre-exposure prophylaxis; PnR, HIV-PrEP-to-need ratio

## Discussion

An estimated 23,644 individuals were prescribed TDF/FTC for HIV-PrEP across nine Canadian provinces in 2021, corresponding to an estimated HIV-PrEP prevalence of 66.9 per 100,000 persons, representing a 1.8-fold increase since 2018. HIV-PrEP uptake varied by age, sex and province. The overall PnR in Canada was 17; however, females, individuals aged 0–19 years, and those in MB, SK and PE had lower levels of HIV-PrEP use relative to epidemic need.

HIV-PrEP use is much higher among males, likely, in part, due to the high uptake of HIV-PrEP among gbMSM. For example, among the 511 individuals accessing HIV-PrEP in AB at sexually transmitted infection, sexual and reproductive health clinics and private family practitioner offices, 98.4% were men and 89.8% were gbMSM ((22)). In addition, challenges encountered by clinicians in identifying women who have HIV-PrEP indications may contribute to lower uptake among females ((18)).

Considering health care in Canada is distributed provincially, coverage of HIV-PrEP remains complex, with different policies between provinces. Several provinces (e.g., BC, SK, AB, MB and PE) offer HIV-PrEP at no cost for those who meet eligibility guidelines and have applicable residence and citizenship status. However, implementation of these programs occurred at different times, and increases in HIV-PrEP uptake and PnR may vary according to increased accessibility to HIV-PrEP. For example, BC has the highest HIV-PrEP prevalence and PnR, and this may be because the HIV-PrEP program is free of charge and has been operating since 2018 ((23,24)). Other provinces provide HIV-PrEP coverage through multiple programs, which sometimes include eligibility criteria and co-payments. This could potentially be contributing to low HIV-PrEP prevalence and PnR, since individuals need to pay for part or all of the entire cost of treatment if they do not have private insurance ((25)). These policy differences between provinces, which are difficult to measure, may account for differences in HIV-PrEP uptake and resulting PnR. This could include the organization and delivery of HIV-PrEP programs, the number of HIV-PrEP providers and access to linguistically and culturally appropriate care ((26)). In addition to these policy differences, further work is needed to examine province-specific challenges in HIV-PrEP uptake.

### Limitations

There are several limitations in our study. The results do not reflect the complete national picture of HIV-PrEP use in Canada, although these nine provinces represented 88.1% of the Canadian population in 2021 ((21)). The addition of information from AB and three territories would provide a more representative overview of HIV-PrEP uptake in Canada. IQVIA data only included prescriptions that were acquired from a community pharmacy. Dispensations from hospital pharmacies, medications provided at no cost and medications purchased online were not included. The dispensation data from IQVIA covered approximately 60% of all retail pharmacies in Canada. Patient counts from participating pharmacies were projected to the whole population of each province by IQVIA and the algorithm used to project dispensations is proprietary. Dispensation data do not include information on medical indication; therefore, an algorithm was used to assign a treatment indication to each dispensation. Although the algorithm for classifying TDF/FTC users as HIV-PrEP users has been validated using data from the United States, it is possible that some dispensations may have been misclassified, and the algorithm may not perform the same in the Canadian context. Not all dispensed prescription drugs are consumed, as some people may have filled a prescription but may not have consumed the medication. These limitations could result in an under- or over-estimate of HIV-PrEP use. This study could not control potential confounders or consider effect modifiers because the database only included limited demographic information. The COVID-19 pandemic has reduced demand for and access to services and has an impact on HIV-PrEP uptake and new HIV diagnoses.

The calculation of PnR was based on new HIV diagnoses, which does not necessarily represent all incident HIV cases. For the PnR, the numerator (number of HIV-PrEP users) could influence the denominator (new HIV diagnoses). Change in overall HIV incidence has been found to be correlated with an increase in PnR ((27)). However, modelling data showed that this impact is likely limited ((28)). Compared to a baseline scale-up scenario of 10% HIV-PrEP coverage, a scale-up scenario of 30% HIV-PrEP coverage reduces HIV incidence over a ten-year period by an estimated 25% ((28)). Modest provision of HIV-PrEP has little substantial impact on new HIV diagnoses. However, if HIV-PrEP is brought to a greater scale, the PnR calculation may need further refinement, such as inclusion of HIV incidence, rather than diagnoses ((19)).

To determine the need for HIV-PrEP use in a particular group of individuals, the World Health Organization uses a ‘substantial risk’ threshold at the group level. Groups with an HIV incidence greater than 3 per 100 person-years are considered at risk and should be recommended for HIV-PrEP ((29)). Unfortunately, the additional sociodemographic variables are not available through the IQVIA administrative pharmaceutical dataset used to estimate HIV-PrEP update and, therefore, could not estimate PnR by key populations disproportionately impacted by HIV in Canada.

## Conclusion

In Canada, the use of HIV-PrEP increased from 2018–2021, however, uptake varied by age, sex and geography. The PnR attempts to provide an opportunity for comparisons regarding whether HIV-PrEP coverage reflects the need for prevention ((20)). HIV-PrEP-to-need ratio may be a useful measure to report on the use of HIV-PrEP as a prevention strategy and can be used to explore disparities in provision across jurisdictions and available demographic categories. As well, this type of measure could be used to help inform program planning and policies for other similar diseases (e.g., Doxy-HIV-PrEP for bacterial sexually transmitted diseases).
